# Atomistic Investigation of Doping Effects on Electrocatalytic Properties of Cobalt Oxides for Water Oxidation

**DOI:** 10.1002/advs.201801632

**Published:** 2018-10-18

**Authors:** Byunghoon Kim, Inchul Park, Gabin Yoon, Ju Seong Kim, Hyunah Kim, Kisuk Kang

**Affiliations:** ^1^ Department of Materials Science and Engineering Research Institute of Advanced Materials (RIAM) Seoul National University 1 Gwanak‐ro Gwanak‐gu Seoul 151‐742 Republic of Korea; ^2^ Center for Nanoparticle Research Institute for Basic Science (IBS) Seoul National University 1 Gwanak‐ro Gwanak‐gu Seoul 151‐742 Republic of Korea

**Keywords:** cobalt oxide, electrolysis, nonprecious transition metal oxide catalysts, oxygen evolution reaction, water splitting

## Abstract

The development of high‐performance oxygen evolution reaction (OER) catalysts is crucial to achieve the clean production of hydrogen via water splitting. Recently, Co‐based oxides have been intensively investigated as some of the most efficient and cost‐effective OER catalysts. In particular, compositional tuning of Co‐based oxides via doping or substitution is shown to significantly affect their catalytic activity. Nevertheless, the origin of this enhanced catalytic activity and the reaction mechanism occurring at catalytic active sites remain controversial. Theoretical investigations are performed on the electrocatalytic properties of pristine and transition metal (Fe, Ni, and Mn)‐substituted Co oxides using first‐principle calculations. A comprehensive evaluation of the doping effects is conducted by considering various oxygen local environments in the crystal structure, which helps elucidate the mechanism behind the doping‐induced enhancement of Co‐based catalysts. It is demonstrated that the local distortion induced by dopant cations remarkably facilitates the catalysis at a specific site by modulating the hydrogen bonding. In particular, the presence of Jahn–Teller‐active Fe(IV) is shown to result in a substantial reduction in the overpotential at the initially inactive catalysis site without compromising the activity of the pristine active sites, supporting previous experimental observations of exceptional OER performance for Fe‐containing Co oxides.

## 1 Introduction

Electrochemical water splitting provides a sustainable means of generating hydrogen fuel, a carbon‐free alternative to fossil fuels.^1^, ^2^, ^3^, ^4^ The bottleneck in the water‐splitting reaction is the sluggish kinetics of the oxygen evolution reaction (OER), which is attributed to its complex four‐electron pathway.^5^, ^6^, ^7^ Although precious‐metal‐oxide‐based materials such as IrO_2_ and RuO_2_ deliver superior catalytic activity toward the OER, their scarcity and high cost have prohibited their large‐scale commercialization.^8^, ^9^ In this respect, earth‐abundant, first‐row (3d) transition metal oxides have recently attracted great interest.^10^, ^11^, ^12^ Amorphous cobalt oxide films in particular have generated substantial research interest because of their cost‐effectiveness, moderate efficiency in neutral electrolytes, and self‐repairing capability.^13^, ^14^ An X‐ray absorption spectroscopy (XAS) study revealed that amorphous cobalt oxide consists of cuboidal Co_4_O_4_ building units, i.e., edge‐sharing CoO_6_ octahedra, with a structure similar to that of the Mn_4_Ca complex of the photosynthetic system, a key component in the biological OER process, thus partly explaining its excellent catalytic activity.^15^ In addition to Co oxides, other 3d metal oxides with similar structural motifs have also been extensively explored.^12^, ^16^, ^17^, ^18^ However, the catalytic activity of these catalysts are inferior to those of Co oxides, and the turnover frequencies are still several orders lower than that of the Mn_4_Ca complex.^19^

As an effective strategy to enhance the activity of 3d metal–based catalysts, great efforts have recently been dedicated to multimetal systems.^20^ The exploitation of the large diversity of metal compositions has led to the discovery of various multimetal‐oxide‐based catalysts, including Ni–Fe,^21^, ^22^, ^23^ Co–Fe,^24^, ^25^, ^26^ Ni–Co,^27^, ^28^, ^29^ Co–Mn,^30^, ^31^ and ternary oxides/oxyhydroxides.^32^, ^33^, ^34^ Of these materials, Fe‐containing Co and Ni oxide systems are known to be the most active toward the OER in alkaline electrolytes.^21^, ^22^, ^23^, ^24^, ^25^, ^26^, ^33^ The introduction of Fe has been shown to enhance the intrinsic activity of Co and Ni oxyhydroxides by ≈100‐fold and 500‐fold, respectively.^23^, ^24^ Many studies have been conducted to clarify the origin of the improved activity of Fe‐containing Co‐ and Ni‐oxide‐based catalysts. For example, it has recently been reported that in Co–Fe oxyhydroxide, the Fe sites are the primary catalytic active sites while the Co oxide serves as an electrically conductive host.^24^, ^26^ However, other researchers have argued that the presence of Fe stabilizes the higher oxidation levels of Co and Ni ions in binary or ternary Co–Ni–Fe oxides, thereby possibly changing the rate‐determining step.^33^ In addition to the debatable roles of the dopants, accurate determination of the catalytic active sites and their reaction mechanism remain elusive. An atomistic‐scale understanding of the active sites would enable more precise estimation and prediction of the intrinsic activity of catalysts. However, to date, atomistic‐scale investigations of multimetal‐oxide‐based catalysts have focused solely on Ni‐based multicatalysts.^35^, ^36^, ^37^

Herein, we present a theoretical study on the electrocatalytic properties of pristine and transition metal (Fe, Ni, and Mn)‐substituted Co oxides. The active sites and reaction mechanism are predicted from extensive examination of the oxygen sites near the dopants. The OER mechanisms are discussed in detail for each site, including the terminal/bridge oxygen sites neighboring the dopant or Co cations in the structure. Notably, it is demonstrated that the bridge oxygen site, which has generally been considered to be inactive for the OER, can be catalytically activated by Fe incorporation. We further unveil the structural properties of the relevant intermediates and propose that the modulation of the hydrogen bonding length induced by the Jahn–Teller‐active Fe(IV) cation leads to the activation of the bridge site. Finally, with a comprehensive view of the estimated OER thermodynamics, we discuss the origin of the improved OER performance of Fe‐containing Co‐oxide‐based catalysts. Our findings help establish theoretical design rules for Co‐based multimetal‐oxide‐based catalysts, and we propose a viable strategy to resuscitate the catalytic activity of inert sites via structural modulation using cation doping.

## 2 Results and Discussion

**Figure 1:**
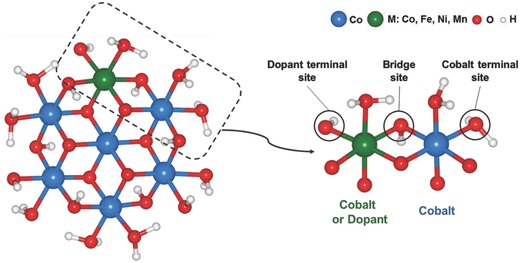
(Left) Pristine or metal‐doped Co oxide cluster model with the composition Co_6_MO_24_H_27_ (M: Co, Fe, Ni, Mn). (Right) The part of the cluster including the oxygen sites considered for the OER mechanism analysis (enclosed by the dotted rectangle on the left side). The cobalt terminal site is the terminal oxygen bound to the Co ion, placed in the second coordination sphere of the dopant. The dopant terminal site is the terminal oxygen bound to the doped metal. The bridge site is the μ_2_‐O connecting the doped metal and Co ion.

An oxide cluster model was adopted in our theoretical investigations on the catalytic mechanism for both the pristine and doped Co oxides, as shown in **Figure** **1** (see the Supporting Information for details of the model construction).^38^ The metal‐doped Co oxides were modeled by substituting one of the outer Co atoms in the cluster with Fe, Ni, or Mn. The change in the catalytic properties was mainly probed near the active sites around the dopant.^39^, ^40^ We also verified that the effect of dopants on the catalytic activities of oxygens bound to second nearest metals or farther metals is negligible (see Figure S4 in the Supporting Information). In this respect, three representative oxygen sites near the dopant, i.e., cobalt terminal oxygen, dopant terminal oxygen, and bridge oxygen sites, were considered to evaluate the doping effects on the OER properties, as illustrated in the right panel of Figure 1. In the figure, the cobalt terminal oxygen refers to the terminal oxygen (η‐O) bound to Co placed in the second coordination sphere of the dopant, whereas the dopant terminal oxygen is the terminal oxygen bound to the dopant cation, and the bridge oxygen (μ_2_‐O) is the oxygen that bridges the doped metal and Co. The acid–base mechanism was considered to involve four elementary steps of the OER, where the intermediates OH*, O*, and OOH* are formed in sequence through the four proton‐coupled electron transfer (PCET) steps (see the Supporting Information for details).^41^, ^42^

**Figure 2:**
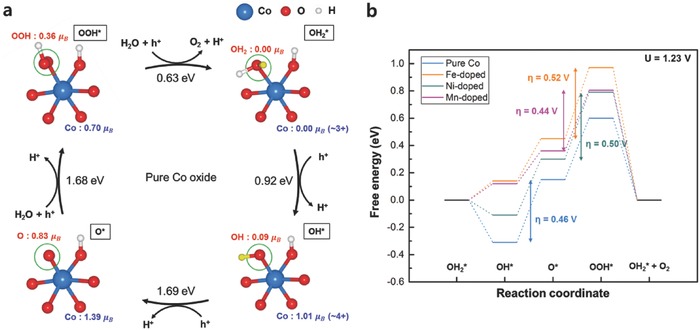
a) OER cycles for the cobalt terminal site of pristine Co oxide. The reaction sites are denoted by green circles, and the H atoms to be removed in the next step are shown in yellow. The integrated spin moments of the species are also represented in units of electron spin (μ_B_). The spin moments data calculated for the cobalt terminal sites of other metal‐doped models are listed in Table S2 (Supporting Information). b) Free energy diagram for the OER of the cobalt terminal sites at 1.23 V (ideal potential for OER). The blue, orange, gray, and purple lines indicate the relative free energies of pristine, Fe‐, Ni‐, and Mn‐doped Co oxides, respectively.

A schematic illustration of the OER on the cobalt terminal site (green circle) of the pristine cobalt oxide is displayed in **Figure** **2**a. Starting from the top right, the formation of the intermediates in the four OER steps is represented along with the integrated spin moments of the species in units of electron spin (μ_B_) to probe the electron transfer at each step. During the first two PCET steps, the holes required for O—O bond formation are accumulated in the terminal Co=O group. The initial extraction of one H^+^ and e^−^ from the terminal Co—OH_2_ group oxidizes Co(III) to Co(IV), preceding the formation of the terminal Co—OH group. The removal of another H^+^ and e^−^ further oxidizes the terminal Co—OH group, yielding a Co=O group with η‐oxo ligand. The two holes produced by the first two oxidation steps are accommodated in both the Co ion and η‐oxo ligand, as observed by the change in the spin moments of each species (from 0.00 to 1.39μ_B_ for Co and from 0.00 to 0.83μ_B_ for O) in Figure 2a as well as the charge density plot in Figure S6 (Supporting Information). This observation indicates that the valence states of the Co and O ions in the terminal Co=O group are +4 and −1, respectively. This formation of the Co(IV)—O• oxyl radical state is consistent with the results of previous theoretical studies on Co oxide,^43^, ^44^ and experimental observations of Co(IV) species.^15^, ^45^ In addition, such a Co(IV)—O• oxyl radical state has been suggested to be a reactive precursor for O—O bond formation.^43^, ^44^ The terminal Co(IV)—O• group is then subject to the nucleophilic attack of water during the third PCET step, forming a O—O bond in the terminal Co—OOH group. Finally, the terminal Co—OOH group releases an O_2_ molecule, completing the OER cycle with the restoration of the catalyst to its initial state.

The blue line in Figure 2b shows the free energy landscape for the OER occurring at the cobalt terminal sites. Each horizontal line indicates the relative energy level of the corresponding state with respect to the initial state at 1.23 V versus RHE, whereas the vertical line with a specific voltage represents the overpotential (η) for the entire OER process, which is 0.46 V for the pristine Co oxide. The second (OH* → O*) and third (O* → OOH*) steps require similar η values of 0.46 and 0.45 eV, respectively, suggesting that both steps contribute to the thermodynamic limitations of the OER to similar extents. Calculations on the same terminal site with the doping were further performed to probe the change in the catalytic activity at the site. However, the doping of the adjacent Co site with Fe, Ni, or Mn did not significantly affect the catalytic activity around the cobalt terminal sites (see Figures S7–S9 and Table S2 in the Supporting Information for the OER cycles on the cobalt terminal site for Fe‐, Ni‐, and Mn‐doped systems). The four lines in Figure 2b compare the OER energy steps occurring at the cobalt terminal sites with and without the dopant in the second coordinate sphere. The η values estimated for the Fe‐, Ni‐, and Mn‐doped systems were ≈0.52, 0.50, and 0.44 V, respectively, which are close to value of 0.46 V for pristine Co oxide. Distinct from the pristine Co case, doping with Fe, Ni, and Mn makes the third step the sole potential‐determining step, along with a slight decrease of the energy required for the second step. However, the similar values of the overall η for the doping of Fe, Ni, and Mn compared with the pristine case indicate that the dopants did not greatly affect the OER activities at the cobalt terminal sites.

**Figure 3:**
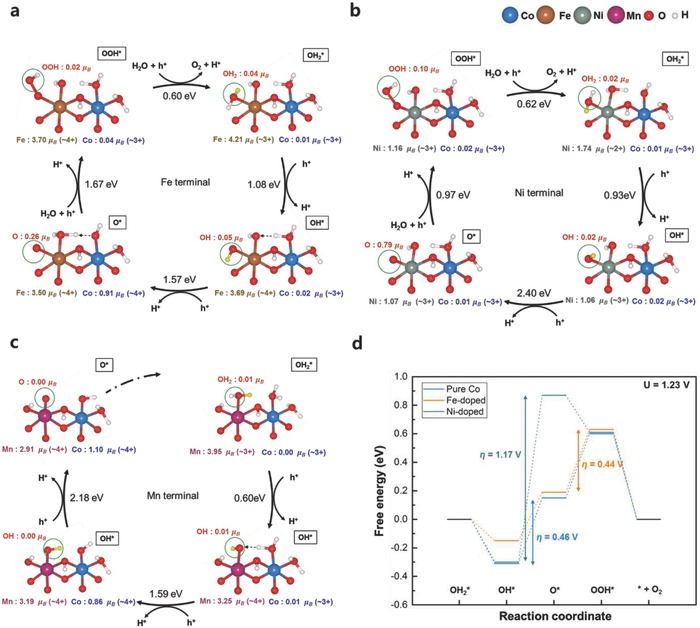
OER cycles for the a) Fe, b) Ni, and c) Mn terminal sites. The reaction sites are denoted by green circles, and the H atoms to be removed in the next step are colored yellow. The integrated spin moments of the species are represented in units of electron spin (μ_B_). d) Free energy diagram for the OER of the dopant terminal sites at 1.23 V (ideal potential for the OER). The blue, orange, and gray lines indicate the relative free energies of the pristine, Fe‐doped, and Ni‐doped Co oxides, respectively.

We further investigated the OER process occurring at the dopant (Fe, Ni, or Mn) terminal sites, as shown in **Figure** **3**. Figure 3a depicts the OER cycles predicted for the Fe terminal oxygen site (green circle) in the Fe‐doped system. The overall OER process at the Fe terminal site was generally similar to that at the cobalt terminal site described above; however, a notable change in the redox center during the PCET steps was observed, particularly in the second step (OH* → O*). The first step (starting from the top right) commences with the removal of H^+^ from the terminal Fe—OH_2_ group, which is accompanied by the oxidation of Fe(III) to Fe(IV). In the second step, while the terminal Fe—OH group is further deprotonated to form a η‐oxo ligand, one proton is transferred from the neighboring Co—OH_2_ to the other Fe terminal oxygen adjacent to the reaction site, as indicated by the black dotted arrow in Figure 3a. As a result, the neighboring Co(III) ion, whose terminal site is deprotonated, is oxidized to Co(IV). This observation contrasts with that for the pure Co case, where the η‐oxo ligand was solely responsible for the oxidation in the second step. This difference is attributable to the highly electrophilic character of the Fe(IV)‐oxo group, which makes accepting more holes difficult,^46^ suggesting that the neighboring Co ion facilitates the catalysis at the Fe terminal site by serving as a hole reservoir. In the subsequent steps, the nucleophilic attack of water occurs at the η‐oxo site, followed by the desorption of the O_2_ molecule. For the Fe‐doped system, the O—O bond formation step was observed to be the potential‐determining step, requiring a theoretical η of 0.44 V, which is similar to that at the cobalt terminal site, 0.46 V, as shown in Figure 3d. Notably, the activity of the Fe site was remarkably enhanced in the Co oxide host structure compared with that in the pure Fe oxide/hydroxide. Pure FeOOH is known to exhibit poor catalytic activity toward the OER, and the oxidation of Fe(III) to Fe(IV) has not been detected even when a large overpotential is applied.^47^, ^48^ However, in our case, Fe oxidation during the first PCET step (OH_2_* → OH*) only required a reaction free energy of 1.08 eV, suggesting that the Co oxide host provides an environment in which the higher valence state of Fe can be stabilized. This observation is also consistent with previous analysis on the structurally analogous β‐CoOOH, for which similar theoretical η values of 0.47 and 0.43 V were reported for the pristine and Fe‐doped structure, respectively.^49^

Unlike the observed catalytic activity of the Fe terminal site in the Co oxide host, the Ni terminal site in the same host exhibited negligible catalytic activity. In the initial OH_2_* state of the OER cycle, the doped Ni is in the 2+ valence state because of the charge transfer between Ni and Co (see Figure 3b). As shown in Figure S10 (Supporting Information), the charge transfer from Co^3+^ to Ni^3+^ yields the Ni^2+^ and Co^4+^ configuration, which is 0.38 eV more stable than the Ni^3+^ and Co^3+^ configuration. This charge transfer from Co to Ni is consistent with the findings of previous in situ XAS and cyclic voltammetry analyses.^50^ The Ni terminal site changes from Ni(II)—OH_2_ to Ni(III)—OH, Ni(III)—O•, and Ni(III)—OOH groups in turn during the OER cycle. Of the four PCET steps, the second step (OH* → O*) is estimated to be potential‐determining, delivering a theoretical η of 1.17 V (Figure 3d). This finding indicates that the activity of the Ni terminal site is mainly limited by the large overpotential required for the O* formation. Similarly, the Mn terminal site is also observed to be inactive in the Co oxide host structure. Although the first step requiring a reaction free energy of 0.60 eV is not demanding, the subsequent deprotonation from the terminal Mn—OH group to produce the terminal Mn=O group requires unusually high energy in the following steps (Figure 3c). Indeed, the deprotonation from the Mn terminal site results in spontaneous proton transfer from the adjacent terminal Co—OH_2_ group to recover the terminal Mn—OH state, preventing progress from the OH* to O* state at the Mn terminal site (denoted by the black dotted arrow in the bottom right panel in Figure 3c). Consequently, the removal of an additional proton–electron pair from the terminal Mn—OH group is necessary to form the η‐oxo suitable for the adsorption of water and consequent O—O bond formation, with an additional free energy of 2.18 eV required for this additional oxidation step. The difficulty in forming the O* state is thought to severely deactivate the Mn terminal site for the Mn‐doped Co oxide.

**Figure 4:**
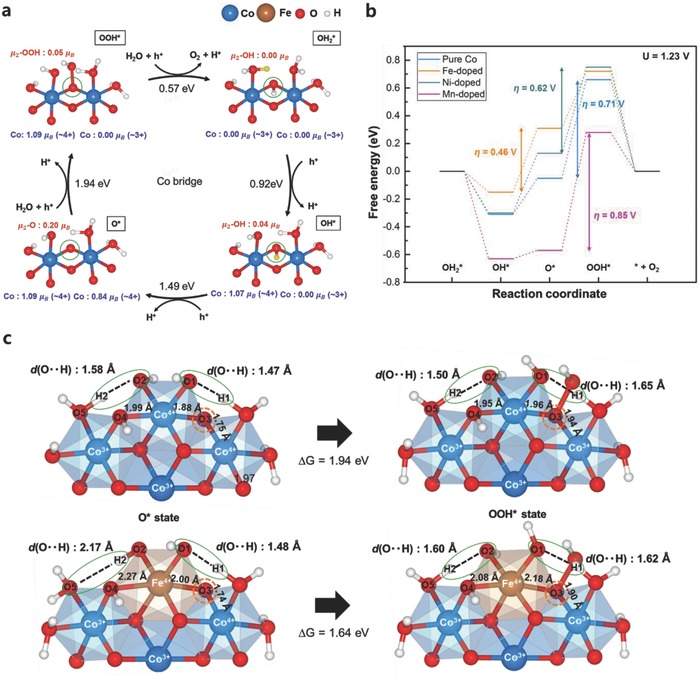
a) OER cycles for the bridge sites of pristine Co oxide. The bridge sites are denoted by green circles, and the H atoms to be removed in the next step are colored yellow. The integrated spin moments of the species are represented in units of electron spin (μ_B_). b) Free energy diagram for the OER of the bridge sites at 1.23 V (ideal potential for the OER). The blue, orange, gray, and purple lines indicate the relative free energies of the pristine, Fe‐, Ni‐, and Mn‐doped Co oxides, respectively. c) Comparison of the structures of pristine and Fe‐doped Co oxides for the O* → OOH* step. The reaction sites are represented by orange circles, and the hydrogen bonds around the active sites are indicated by green circles. Only half of the cluster model is described for clarity.

Finally, the bridge oxygen sites were investigated as potential catalytic sites. Although the bridge sites have generally been regarded as inactive for the OER, it has recently been proposed that the bridge site could participate in the OER under certain conditions.^44^, ^51^, ^52^ A comparative study on the OER properties of Co oxide and Co phyllosilicate indicated that the introduction of specific anion groups (i.e., a phyllosilicate group) onto the Co oxide could make the bridge site a major active site.^51^ In addition, density functional theory (DFT) calculations on RuO_2_ indicated that the incorporation of Ni or Co into RuO_2_ could involve the bridge site in the OER process as a proton acceptor of neighboring active sites.^52^ In this regard, we closely examined the catalytic activity at the bridge site in the doped Co oxides. The OER process on the bridge site generally involves the redox of two neighboring metal ions. For pure Co oxide, for example, two holes (or electrons), which are generated from the first (or the last) two PCET steps, reside on two separate Co ions for the OER cycle on the bridge site, as shown in **Figure** **4**a (see Figure S11 in the Supporting Information for the charge density plot). The OER pathways on the bridge site of Fe‐, Ni‐, and Mn‐doped systems proceed in a similar way, and the redox centers are shared by both Co and the doped metal (see Figures S12–S14 in the Supporting Information). Figure 4b compares the OER energies estimated for the bridge sites for the pristine and doped Co oxides. For the pure Co oxide, the activity of the bridge site is limited by the large overpotential of 0.71 V. Similar values were obtained for the Ni‐ and Mn‐doped systems. However, a significant reduction in the overpotential was observed for the Fe‐doped system with a theoretical η of 0.46 V, which suggests the Fe bridge site as a new OER active site.

To understand the catalytic activity at the bridge site in the Fe‐doped system, its structural properties were carefully examined. Although the OOH* formation step was the determining step for pristine Co oxides (1.94 eV), the introduction of Fe significantly reduced the energy for the equivalent step to 1.64 eV, indicating that the activation of the bridge site in the Fe‐doped oxide is likely to originate from the more stable OOH* adsorption compared with that of the undoped model. We observed that this stabilization is closely related to the structural distortion induced by the Jahn–Teller‐active Fe(IV). Figure 4c shows the atomic configurations around active sites for the O* and OOH* states of the pristine and Fe‐doped Co oxides. One notable difference is the length of the hydrogen bonds around the active sites for the pure (top panel) and Fe‐doped (bottom panel) systems (green circle). In the O* state, an unusually long O—H bond was observed for the Fe‐doped Co oxide, indicating a substantially weaker hydrogen bond than that for the pristine Co oxide. Although the O1—H1 bond length was similar for both systems, the O2—H2 bond (green circle) of the Fe‐doped system (2.17 Å) was far longer than that of the pristine Co oxide (1.58 Å). This difference is attributed to the Jahn–Teller distortion of Fe(IV), where the elongation along the O3—Fe—O4 direction greatly separates O2 and O5 (3.02 Å), which in turn makes it difficult to form a strong hydrogen bond. However, when the OOH* intermediate is formed, the O2—H2 bond is greatly shortened (2.17 → 1.60 Å). Moreover, as the Fe—O3 distance is elongated by the water adsorption at the μ_2_‐O3 site, the Fe—O4 distance decreases, resulting in stronger O2—H2 bonding. This finding suggests that the initial relative instability arising from the weak hydrogen bonding in the O* state could be significantly relieved by the water adsorption in the OOH* state. Although the thermodynamic benefits of this stronger hydrogen bonding formation cannot be expected for pristine Co oxide during the OOH* formation step, this finding suggests that the inherent local distortion of Fe eventually activates the bridge site by virtue of the strengthening of the hydrogen bond. A similar beneficial contribution of structural distortion to OER catalysis has also been observed for cobalt diphosphate, where the distorted metal coordination geometry was found to favor the water adsorption lowering the activation barrier of the O—O bond formation.^53^

**Figure 5:**
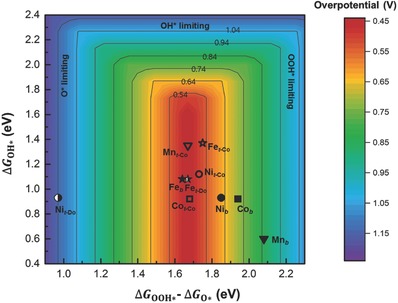
2D contour map showing comprehensive results of metal doping on OER activities. This contour map was constructed by assuming a scaling relation of Δ*G*_OOH*_ = Δ*G*_OH*_ + (3.34 ± 0.10) eV, which was derived from the fitting in Figure S15 (Supporting Information). The square, star, circle, and triangle symbols represent Δ*G* of the pristine, Fe‐, Ni‐, and Mn‐doped Co oxides, respectively. In addition, the hollow, half‐filled, and filled symbols indicate Δ*G* of the cobalt terminal sites (denoted by the subscript “t‐Co”), dopant terminal sites (denoted by the subscript “t‐Do”), and bridge sites (denoted by the subscript “b”), respectively.

The resulting overpotentials of all the reaction sites considered for the pristine and doped Co oxides are represented in the 2D map of Δ*G*_OOH*_ − Δ*G*_O*_ and Δ*G*_OH*_ in **Figure** **5**. This contour map was constructed by assuming the scaling relation of Δ*G*_OOH*_ = Δ*G*_OH*_ + (3.34 ± 0.10) eV (see Figure S15 in the Supporting Information for further detail), which indicates only a small discrepancy in the intercept value with an approximate universal scaling relation of Δ*G*_OOH*_ = Δ*G*_OH*_ + (3.2 ± 0.20) eV estimated for rutile oxides and perovskites.^42^ Overall, the activity of the cobalt terminal sites (hollow symbols) is not highly dependent on the dopant type, but the activity of the dopant terminal sites (half‐filled symbols) and bridge sites (filled symbols) varies considerably with dopant types. Among the doped metals, Fe is particularly beneficial for the overall catalytic efficiency. Notably, the terminal oxygen sites, which are considered the main active sites in pristine Co oxides, are not responsible for the enhanced OER performance of Fe‐doped Co oxides. The cobalt terminal site (denoted as Fe_t‐Co_) and dopant terminal site (denoted as Fe_t‐Do_) in the Fe‐doped model exhibited similar theoretical overpotentials as the terminal site of pristine Co oxide (denoted as Co_t‐Co_). However, the activity of the bridge site was remarkably enhanced in the Fe‐doped Co oxide compared with that in the pristine Co oxide. This finding suggests that the exceptional performance of Fe‐containing Co oxides originates from the provision of additional active sites represented by the bridge site (denoted as Fe_b_) rather than from the further enhancement of the intrinsic activity of existing active sites. Unlike Fe doping, the doping with Ni or Mn is predicted to result in deterioration of the OER performance of Co oxides. Although the effects of Ni and Mn doping on the cobalt terminal sites were modest, the Ni and Mn terminal sites were estimated to be inactive toward the OER. In addition, the catalytic activation of the bridge site is not expected for Ni‐ and Mn‐doped systems. Therefore, the large overpotential of the dopant terminal sites would induce an overall reduction in the number of exposed active sites, resulting in degradation of the OER performance.

Co oxyhydroxides are one of the most representative layered structures along with their lithium analog (LiCoO_2_), and it is well‐known that they typically crystalize in the plate‐like structures in the nature or in the conventional synthetic process.^54^, ^55^, ^56^, ^57^ In this respect, in the present study, we adopted the layered motif to properly simulate the local environment of the reaction sites. Nevertheless, considering the amorphous feature of Co oxides, other cluster models can be also the candidate models describing Co oxides, where the local environment around dopants might be different from that of the present cluster model. In this regard, we investigated the effect of cluster geometry on OER properties using several clusters with altered geometries and configurations, such as a nonflat cluster (see Figures S16–S18 and their related parts in the Supporting Information). It was found that the findings of the present work based on planar cluster models are valid for many other different models, and thus widely applicable. For more exhaustive understanding of doping effects, further study considering more diverse cluster models with different connectivity of oxide sheets is needed, which is the avenue of our future work.

## 3 Conclusion

We investigated the OER mechanism for pristine and (Fe, Ni, and Mn)‐substituted Co oxides using first‐principle calculations. Regardless of the dopant type, the changes in η of the cobalt terminal site were less than 0.1 V. The Fe terminal site exhibited an η of 0.44 V, which was comparable to that of the terminal site of pristine Co oxide (η = 0.46 V), whereas the activity of the Ni and Mn terminal sites was severely limited by difficulties in forming O* intermediates. Notably, the presence of Fe significantly reduced the η at the bridge site from 0.71 to 0.46 V. This catalytic activation of the bridge site was attributed to the stabilization of OOH* formation, which benefits from the local distortion of the Fe cation and consequent dramatic change in the hydrogen bond strength. From these results, we propose that the key origin of the enhanced performance of Fe‐containing Co oxides is the supply of additional active sites represented by the bridge site rather than the improvement of the existing active site denoted as the terminal site. These findings help to clarify the OER performance of recently reported Co‐oxide‐based catalysts and provide theoretical guidelines for the rational design of multimetal‐based catalysts. Moreover, it is newly proposed that applying local strain to the native environment of reaction sites using cation doping could be a viable strategy to enhance the catalytic activity of materials.

## Conflict of Interest

The authors declare no conflict of interest.

## Supporting information

Supplementary
