# Supplementary material for: Atomistic Investigation of Doping Effects on Electrocatalytic Properties of Cobalt Oxides for Water Oxidation
Source: Adv Sci (Weinh). 2018 Oct 18;5(12):1801632. doi: 10.1002/advs.201801632 (PMC6299724; doi:10.1002/advs.201801632)
Supplement: Supplementary file 1 — Supplementary [file ADVS-5-1801632-s001.pdf]

Copyright WILEY-VCH Verlag GmbH & Co. KGaA, 69469 Weinheim, Germany, 2018.

ADVANCED SCIENCE
Open Access

Supporting Information

for *Adv. Sci.,* DOI: 10.1002/advs.201801632

Atomistic Investigation of Doping Effects on Electrocatalytic Properties of Cobalt Oxides for Water Oxidation

*Byunghoon Kim, Inchul Park, Gabin Yoon, Ju Seong Kim, Hyunah Kim, and Kisuk Kang**

Copyright WILEY-VCH Verlag GmbH & Co. KGaA, 69469 Weinheim, Germany, 2018.

Supporting Information

Title Atomistic investigation of doping effects on electrocatalytic properties of cobalt oxides for water oxidation

Byunghoon Kim,[†,§] *Inchul Park,*[†,§] *Gabin Yoon,*[†] *Ju Seong Kim,*[†] *Hyunah Kim,*[†] *and Kisuk Kang*[*,†,‡]

[†]Department of Materials Science and Engineering, Research Institute of Advanced Materials (RIAM), Seoul National University, 1 Gwanak-ro, Gwanak-gu, Seoul 151-742, Republic of Korea.

[‡]Center for Nanoparticle Research, Institute for Basic Science (IBS), Seoul National University, 1 Gwanak-ro, Gwanak-gu, Seoul 151-742, Republic of Korea.

Computational details

All the calculations presented in this work were performed based on density functional theory (DFT) using the Vienna *ab initio* simulation package (VASP).[1] The projector augmented wave (PAW) pseudopotentials were used as implemented in VASP. [2] To describe the exchange and correlation energies, the spin-polarized generalized gradient approximation (GGA) parameterized using the Perdew–Burke–Ernzerhof (PBE) functional was utilized along with the Hubbard-*U* correction.[3, 4] Effective Hubbard-*U* parameters of 3.5, 3.5, 5.5, and 3.9 eV, which were fitted to describe experimental parameters in other studies[5,6],were applied to the 3*d* electrons of Co, Fe, Ni, and Mn, respectively. Previous studies have reported similar Hubbard-*U* values, calculated from the linear response theory or fitting experimental parameters. [7] The choice of Hubbard-*U* parameters in the present study can be also justified by noting that the variations in *U* values (-0.3 ~ +1.0 eV from the values used) insignificantly changes the overpotential values (see Figure S3). It has been reported that solvation effects

can play significant roles in the electrochemical reactions that occur at the electrode–electrolyte interface.[8, 9] Thus, solvation effects were also considered using an implicit solvation model using the Vaspsol software package.[10] Periodic cubic supercells with dimensions of 20 Å × 20 Å × 15 Å, wherein a Co oxide cluster was accommodated, were used to secure sufficient vacuum space to make the interactions between the clusters negligible. A plane-wave basis set was used with an energy cutoff of 500 eV, and appropriate k-point meshes were selected to ensure that the total free energy of the system converged within 0.1 meV. To find the correct lowest energy state for each catalytic state, all possible proton and spin configurations were considered.

Thermodynamics of the oxygen evolution reaction

The oxidation of water to generate oxygen, $2H_2O \rightarrow O_2 + 4H^+ + 4e^-$, requires a thermodynamic potential of 1.23 V at standard conditions (T = 298.15 K, P = 1 bar, pH = 0). In practice, however, a substantial overpotential is required for the reaction to proceed at a sufficient rate owing to the sluggish multielectron reactions. For the four elementary proton-coupled electron transfer (PCET) steps of the oxygen evolution reaction (OER) mechanism, the following pathways, which are consistent with the mechanism proposed by Rossmeisl, Nørskov, and coworkers, are considered [11, 12]:

$$OH_2^* + 2H_2O\ (l) \rightarrow OH^* + 2H_2O\ (l) + e^- + H^+ \quad (1)$$

$$OH^* + 2H_2O\ (l) \rightarrow O^* + 2H_2O\ (l) + e^- + H^+ \quad (2)$$

$$O^* + 2H_2O\ (l) \rightarrow OOH^* + H_2O\ (l) + e^- + H^+ \quad (3)$$

$$OOH^* + H_2O\ (l) \rightarrow O_2\ (g) + OH_2^* + e^- + H^+, \quad (4)$$

where * denotes a surface adsorption site.

As the OER proceeds, the intermediates of OH*, O*, and OOH* are formed in sequence. The Gibbs free energy difference for steps (1)–(4) can be calculated using the following equations:

$$\Delta G_1 = \Delta G_{OH*} - eU + \Delta G_{H^+}(pH) \quad (5)$$

$$\Delta G_2 = \Delta G_{O*} - \Delta G_{OH*} - eU + \Delta G_{H^+}(pH) \quad (6)$$

$$\Delta G_3 = \Delta G_{OOH*} - \Delta G_{O*} - eU + \Delta G_{H^+}(pH) \quad (7)$$

$$\Delta G_4 = 4.92 - \Delta G_{OOH*} - eU + \Delta G_{H^+}(pH), \quad (8)$$

where U is the potential measured against a normal hydrogen electrode (NHE) at standard conditions. The Gibbs free energy change of a proton relative to NHE at non-zero pH is expressed by the Nernst equation as $\Delta G_{H^+}(pH) = -k_B T \ln(10) \times pH$. To avoid the calculation including O_2 gas, which is difficult to accurately determine within the generalized gradient approximation–density functional theory (GGA–DFT) scheme, the sum of ΔG_{1-4} is fixed to the negative of the experimental Gibbs free energy of the formation of two water molecules ($2H_2O \rightarrow 2H_2 + O_2$), i.e., 4 × 1.23 = 4.92 eV.[12] The Gibbs free energies of Equation (5)–(8) were determined using the adsorption energies of intermediates such as OH*, O*, and OOH*. The Gibbs free energy differences of each intermediate were calculated from the DFT energy (E_i), zero-point energy (ZPE), and entropy correction using $\Delta G_i = \Delta E_i + \Delta ZPE - T\,\Delta S$. The ZPE was calculated only for adsorbed species at the reaction site, assuming that the ZPE corresponding to the other part of the model does not change during the OER cycle. For the entropy correction, only gas or liquid phase species were considered because the major contribution to the entropy is the translational entropy. The energy difference ΔE_i is calculated relative to H_2O and H_2 at the computational standard hydrogen electrode (SHE), i.e., U = 0 and pH = 0, as

$$\Delta E_{OH*} = E_{OH*} - E_{OH_2*} + \frac{1}{2} E(H_2) \quad (9)$$

$$\Delta E_{O*} = E_{O*} - E_{OH_2*} + E(H_2) \quad (10)$$

$$\Delta E_{OOH*} = E_{OOH*} - E_{OH_2*} - \left[E(H_2O) - \frac{3}{2} E(H_2)\right]. \quad (11)$$

Using the computational SHE allows us to assume that a proton and an electron are in equilibrium with half a hydrogen molecule at U = 0 V *vs.* SHE. [12, 13] The theoretical overpotential does not depend on the potential or pH values because the Gibbs free energy differences in Equations (5)–(8) change in the same way with the potential and pH.

Of the four elementary steps, the step with the largest Gibbs free energy difference limits the overall rate of the reaction and thus become the theoretical potential-determining step (PDS). Therefore, the theoretical overpotential η is defined as

$$\eta = \max[\,\Delta G_1, \Delta G_2, \Delta G_3, \Delta G_4\,]\ /\mathrm{e} - 1.23\ [V].$$

Pure and doped Co oxide cluster model

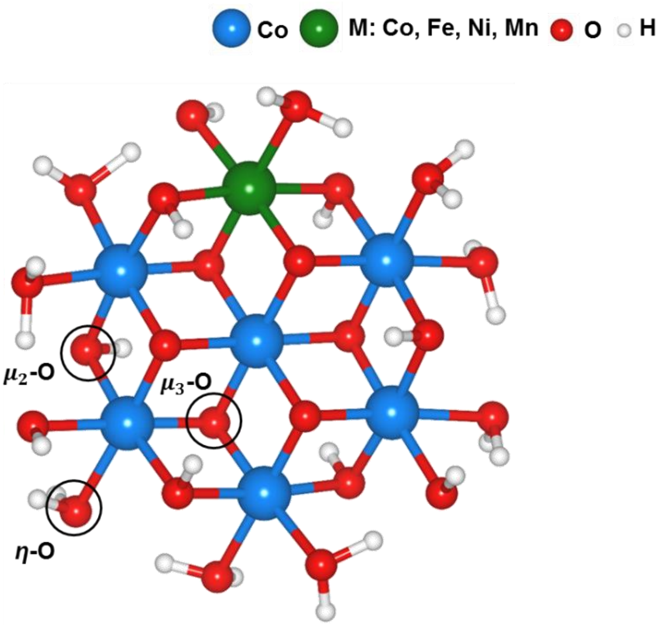

Figure S1. Pristine or metal-doped Co oxide cluster model with the composition $Co_6MO_{24}H_{27}$ (M: Co, Fe, Ni, Mn). Terminal oxygen atoms that do not interconnect metal ions are denoted by η-O. The μ_2-O and μ_3-O atoms interconnect two and three Co ions, respectively.

To compare the catalytic properties of pristine and doped Co oxides, the model of pristine Co oxide should be preferentially defined. A Co oxide cluster model established based on a

detailed comparison between X-ray Absorption Spectroscopy (XAS) analyses and *ab initio* molecular dynamics (AIMD) simulations was applied.[14] The XAS analyses revealed that amorphous Co oxides are composed of blocks of edge-sharing CoO_6 octahedra, although the connectivity between the blocks is difficult to specify because of the amorphous feature of Co oxides. [15] To describe the structure of the edge-sharing CoO_6 octahedra, a Co_7O_{24} cluster was cut out from the Co–O sheet of $LiCoO_2$, a structurally analogous compound to Co oxides, as shown in Figure S1. Because all the Co atoms are surrounded by six O atoms, there are several terminal oxygen atoms that do not interconnect metal ions (labeled "η-O" in Figure 1**)** at the interface between the Co oxide cluster and water. However, μ_2-O and μ_3-O atoms interconnect two and three neighboring metal ions, respectively, as shown in Figure **S**1. The XAS analyses also indicated that Co ions were mainly in the valence state of +3 in the "resting conditions" of the catalyst, which has also been verified by electron paramagnetic resonance spectroscopy analysis. [15-17] As such, the Co oxide cluster was saturated by the appropriate number of H atoms to ensure the valence state of +3 for all Co ions, resulting in a $Co_7O_{24}H_{27}$ cluster. On the basis of the results of AIMD simulations performed by Guidoni *et al.*, all the μ_2-O atoms were protonated, whereas all the μ_3-O atoms were not protonated.[14] The remaining protons were distributed at terminal oxygen sites such that terminal Co–OH_2 and Co–OH groups were formed at the edge sites of the cluster. Terminal Co–O and Co–OH_3 species were not observed in the optimized structure irrespective of the initial position of H atoms, which agrees well with the observations of previous studies.[14] The structure parameters of the optimized Co oxide cluster obtained in the present work were almost identical to those of the equivalent model proposed by Guidoni *et al.* as well as the results of the previous XAS measurements; the deviations of all the considered parameters were less than 0.05 Å, as shown in Figure S2 and Table S1. [14, 16]

The models for the metal-doped Co oxides were designed by substituting one of the outer Co atoms in the aforementioned cluster with Fe, Ni, or Mn (Figure S1). These doped elements

have been experimentally proven to replace Co sites when incorporated into the Co oxide.[18-20] For the description of the resting state of metal-doped models during the OER, the number of saturated H atoms was kept the same as that for pure Co oxide, which ensures the +3 average oxidation state of the metal ions by virtue of the $Co_6MO_{24}H_{27}$ composition. Fe and Mn have been shown to be incorporated into Co oxides in the Fe(III) and Mn(III) state, respectively.[18, 19]Although Ni has been shown to be incorporated into Co oxides in the Ni(II) state, it undergoes the Ni(II) to Ni(III) transition before the onset of the OER when voltage is applied. [20] Our calculation also indicated that the oxidation from $Co_6NiO_{24}H_{28}$ to $Co_6NiO_{24}H_{27}$ required only 0.26 V (Figure S5), such that $Co_6NiO_{24}H_{27}$ was more suitable as a composition for the resting state of the Ni-doped model.

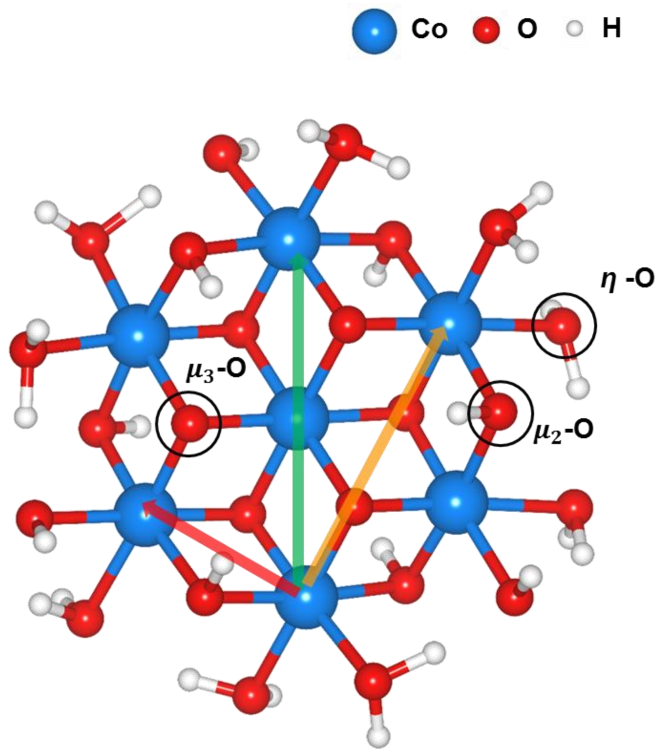

Figure S2. Optimized structure of pristine Co oxide ($Co_7O_{24}H_{27}$) cluster model. Terminal oxygen atoms that do not interconnect metal ions are labeled η-O. The μ_2-O and μ_3-O atoms interconnect two and three Co ions, respectively. The red, orange, and green lines indicate the distance between the first nearest-neighbor Co ions (Co–Co1 in Table S1), second nearest-

neighbor Co ions (Co–Co2 in Table S1), and third nearest-neighbor Co ions (Co–Co3 in Table S1), respectively.

Table S1. Comparison of structure parameters of Co oxides determined using different methods including GGA, GGA+U, GGA+U (Vaspsol), AIMD, and XAS. The bond lengths were averaged over different bonds of the same type. The Co–O (all) term indicates an average over all the Co–O bonds, whereas the Co–O (inner) term indicates the average Co–O bond length over all the bonds except terminal Co–O bonds. The Co–Co1 term is the distance between two neighboring Co atoms interconnected by di-$\mu_{2,3}$-O/OH (marked with the red line in Figure S2). The Co–Co2 term is the distance between second nearest-neighbor Co atoms (marked with the orange line in Figure S2), and the Co–Co3 term is the distance between third nearest-neighbor Co atoms (marked with the green line in Figure S2).

**Method**	**Bond distance/ Å**				
	**Co–O (all)**	**Co–O (inner)**	**Co–Co1**	**Co–Co2**	**Co–Co3**
GGA+U	1.94 ±0.01	1.90±0.04	2.78±0.02	4.82±0.02	5.57±0.01
**GGA+U (Vaspsol)**	**1.94 ±0.01**	**1.90±0.04**	**2.79±0.02**	**4.84±0.02**	**5.59±0.01**
GGA[14]	1.94 ±0.08	1.91 ±0.05	2.79±0.02	4.84±0.02	5.59±0.02
GGA+U[14]	1.93 ±0.07	1.90 ±0.05	2.80±0.02	4.85±0.02	5.60±0.02
AIMD14	1.91 ±0.05	1.91 ±0.05	2.81±0.06	4.86±0.07	5.61±0.07
Co oxide XAS data[16]	1.89	-	2.81	4.86	5.62

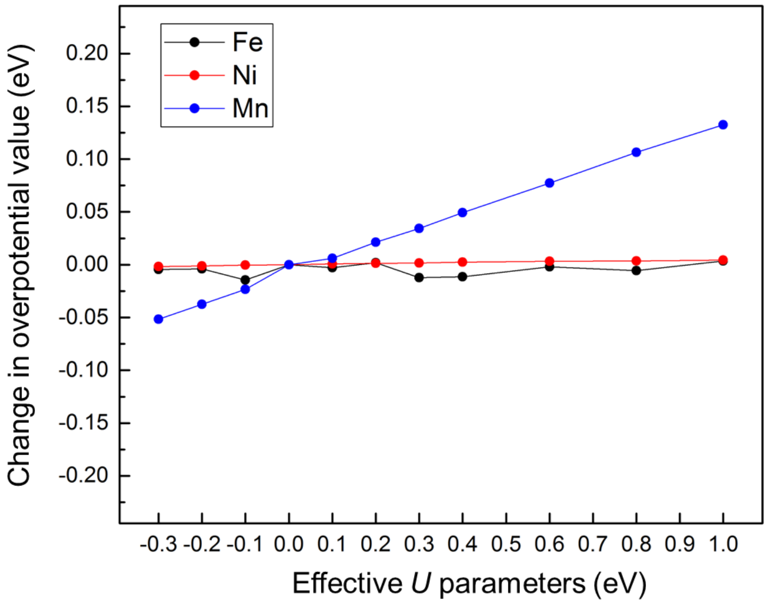

Figure S3. Change in the overpotential value for OER on the bridge site according to variations of effective *U* values. For the OER cycles of Mn-doped models, the variation of the η value is slightly larger with the U values, however, the calculated η is 0.85 V, and this variation does not change the potential-determining step. The conclusion that the reaction sites in Mn-doped model are inactive toward OER is still valid.

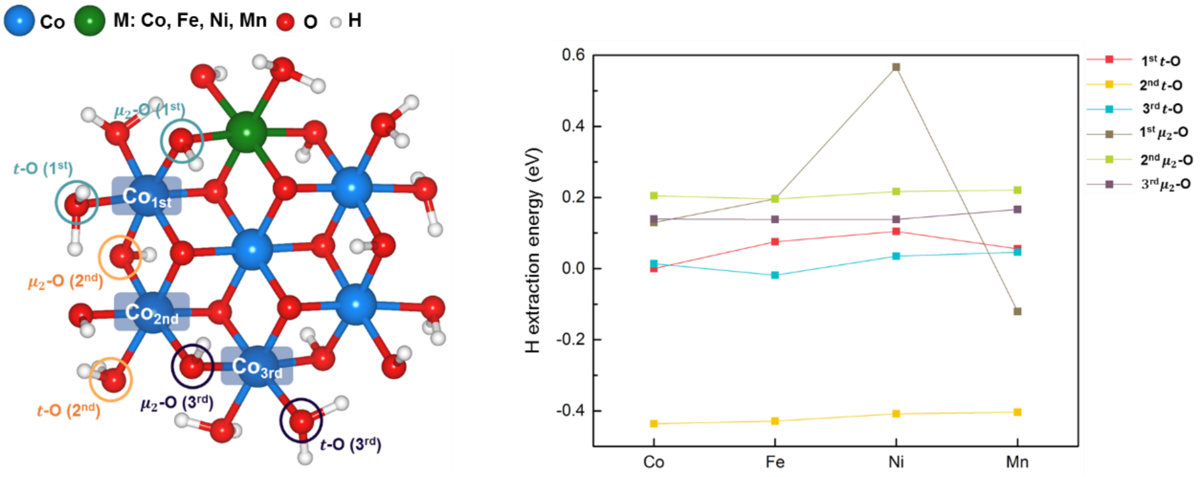

Figure S4. The effect of dopants on the catalytic activities of various oxygen sites. (left) Co_{1st}, Co_{2nd}, and Co_{3rd} indicates the first, second and third closest Co ions to the doped cation, respectively. The terminal and bridge oxygen sites bound to these Co ions are also denoted. (right) The variation of ($H^+ + e^-$) extraction energy depending on the types of dopants. Unlike the case of the first terminal oxygen (1st *t*-O) and bridge oxygen (1st μ_2-O), the hydrogen extraction energies from the second and third oxygen sites are not significantly affected by the type of dopants.

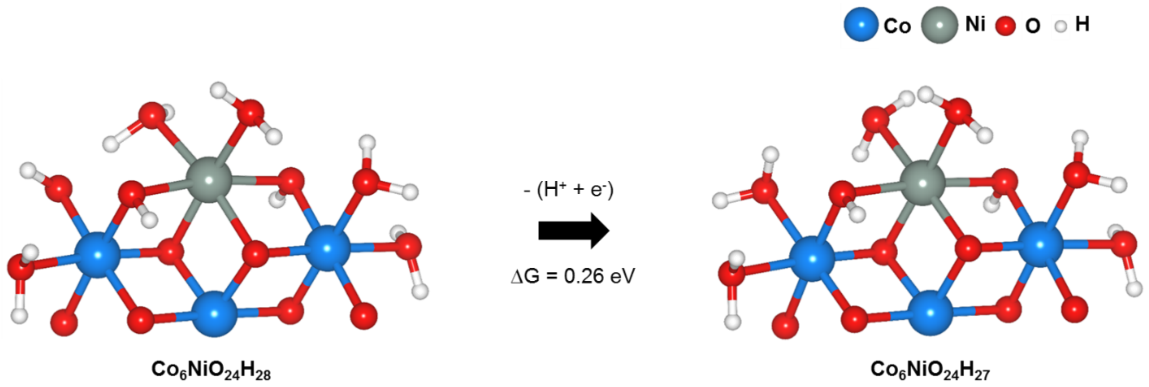

Figure S5. Relaxed structure and Gibbs free energy difference of $Co_6NiO_{24}H_{28}$ and $Co_6NiO_{24}H_{27}$ cluster. Only half of the cluster model is shown for clarity.

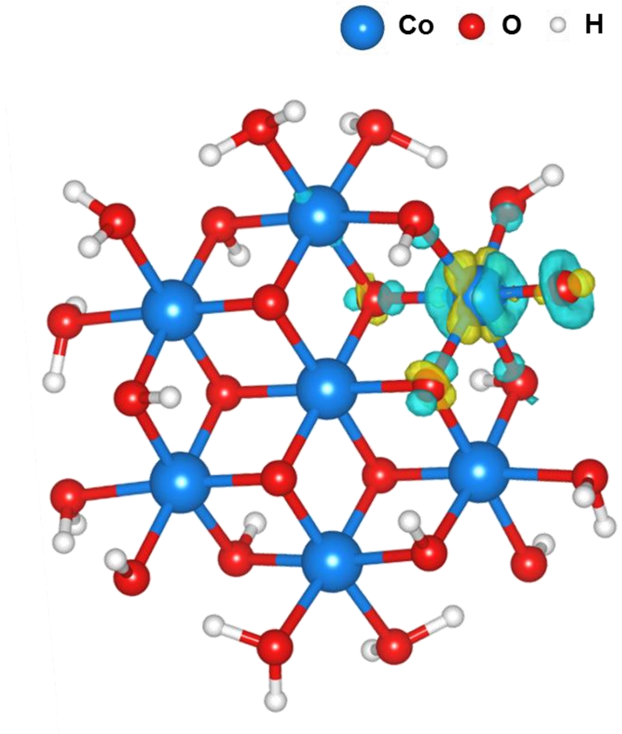

Figure S6. Charge density plot for the terminal site of pristine Co oxide in O* state, showing the location of holes residing in the terminal Co=O group. Blue indicates the charge lost, and yellow indicates the charge gained. The isosurface was set to 0.1 $e^{-}/Å^{3}$.

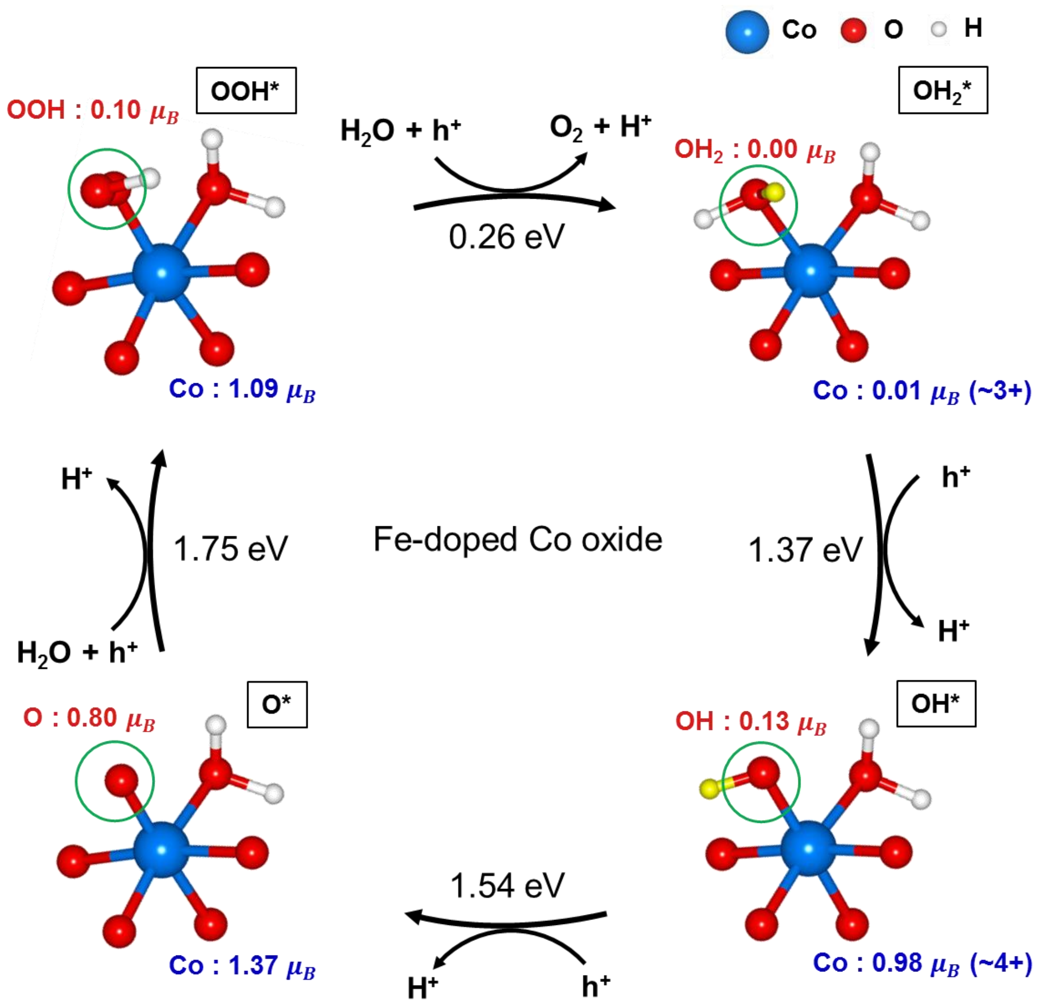

Figure S7. OER cycles for the cobalt terminal site of Fe-doped Co oxide. The H atoms to be removed in the next step are colored yellow. The integrated spin moments of the species are also shown in units of electron spin (μ_B).

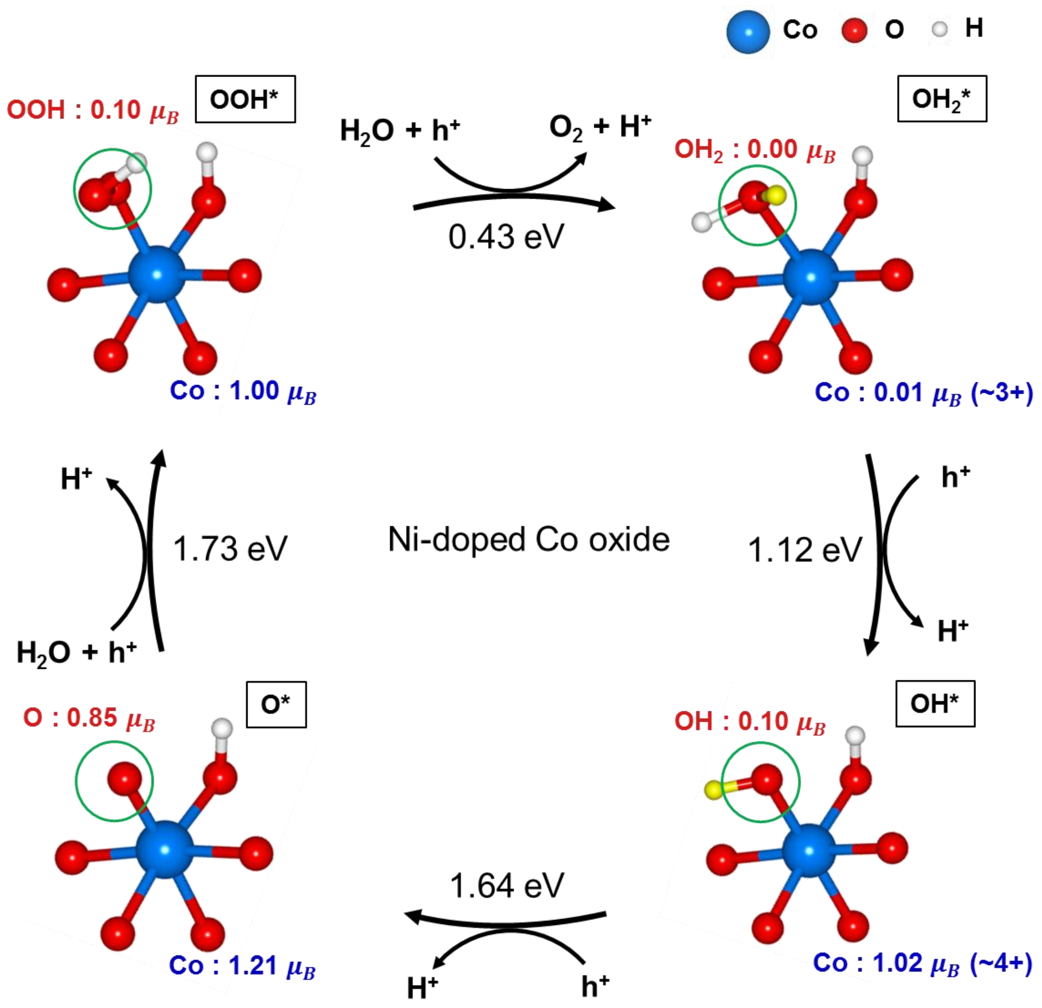

Figure S8. OER cycles for the cobalt terminal site of Ni-doped Co oxide. The H atoms to be removed in the next step are colored yellow. The integrated spin moments of the species are also shown in units of electron spin (μ_B).

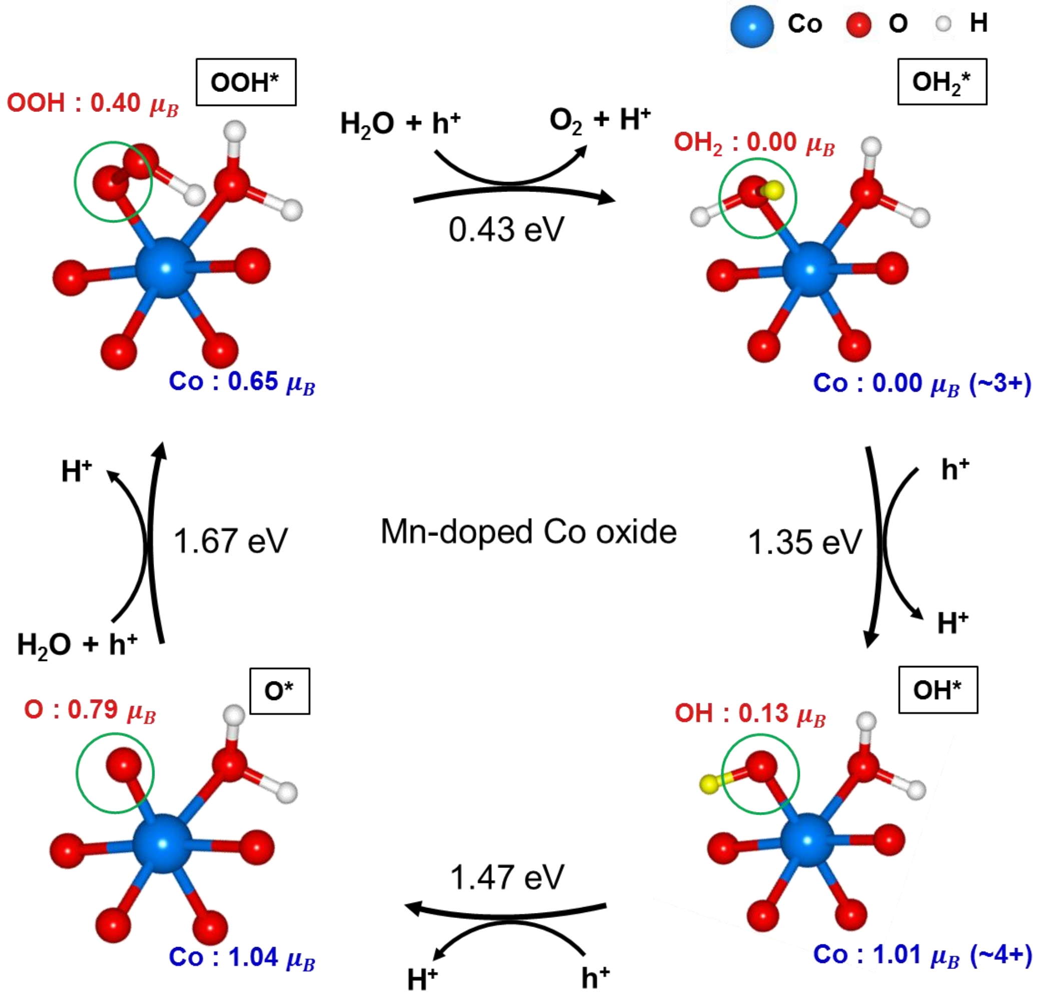

Figure S9. OER cycles for the cobalt terminal site of Mn-doped Co oxide. The H atoms to be removed in the next step are colored yellow. The integrated spin moments of the species are also shown in units of electron spin (μ_B).

Table S2. Calculated spin moments m (in units of μ_B) and Bader charges q (in units of electrons) for the Co ion and adsorbed species on the cobalt terminal sites.

	OH_2* state				OH* state			
	$\lvert m \rvert$: Co	$\lvert m \rvert$: OH_2	q: Co	q: OH_2	$\lvert m \rvert$: Co	$\lvert m \rvert$: OH	q: Co	q: OH
Pure Co	0.00	0.00	+1.40	−1.95	1.01	0.09	+1.56	−1.49
Fe-doped	0.01	0.00	+1.40	−1.95	0.98	0.13	+1.56	−1.48
Ni-doped	0.01	0.00	+1.41	−1.93	1.02	0.10	+1.58	−1.49
Mn-doped	0.00	0.00	+1.40	−1.95	1.01	0.13	+1.56	−1.48
	O* state				OOH* state			
	$\lvert m \rvert$: Co	$\lvert m \rvert$: O	q: Co	q: O	$\lvert m \rvert$: Co	$\lvert m \rvert$: OOH	q: Co	q: OOH
Pure Co	1.39	0.83	+1.60	−0.63	0.70	0.36	+1.51	−1.36
Fe-doped	1.37	0.80	+1.59	−0.64	1.09	0.10	+1.53	−1.43
Ni-doped	1.21	0.85	+1.60	−0.62	1.00	0.10	+1.55	−1.44
Mn-doped	1.04	0.79	+1.56	−0.66	0.65	0.40	+1.48	−1.35

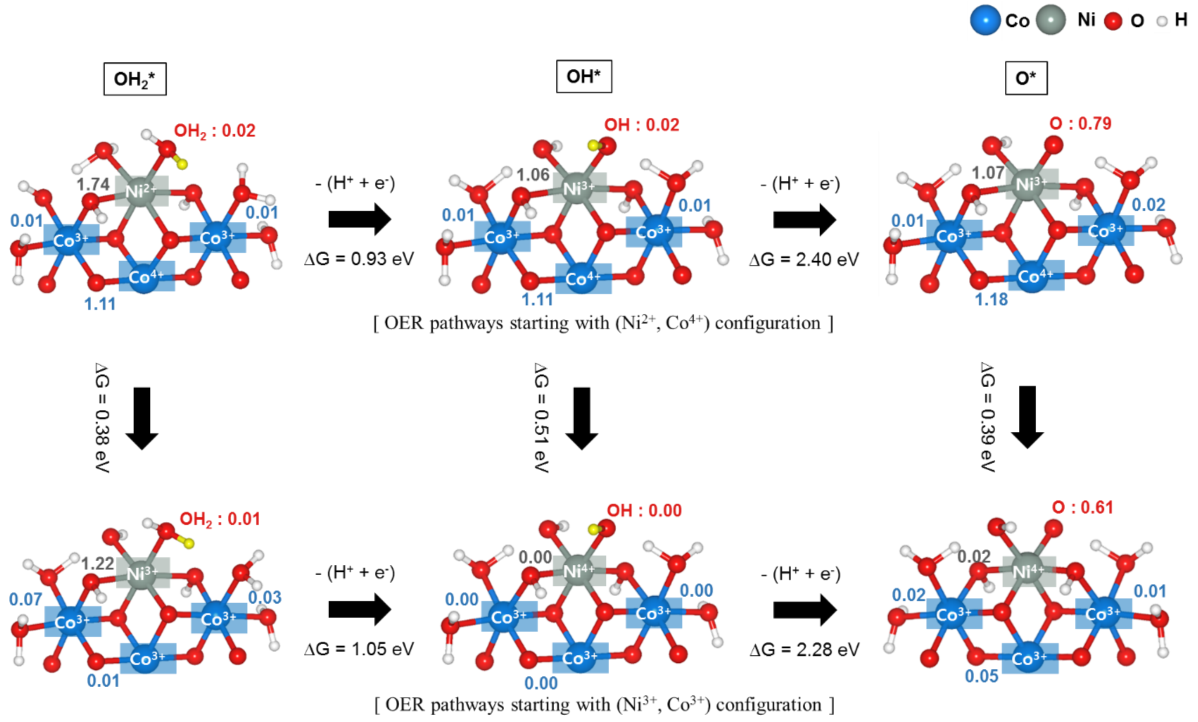

Figure S10. Comparison of OER pathways at the Ni terminal site with (Ni^{2+}, Co^{4+}) and (Ni^{3+}, Co^{3+}) as starting configurations. Only half of the cluster model is shown for clarity. The integrated spin moments of the species are shown in units of electron spin (μ_B).

Table S3. Calculated spin moments m (in units of μ_B) and Bader charges q (in units of electrons) for the metal ions (M) and adsorbed species on the dopant terminal sites.

	OH_2* state				OH* state			
	$\|m\|$: M	$\|m\|$: OH_2	q: M	q: OH_2	$\|m\|$: M	$\|m\|$: OH	q: M	q: OH
Co terminal	0.00	0.00	+1.40	−1.95	1.01	0.09	+1.56	−1.49
Fe terminal	4.21	0.04	+1.91	−1.96	3.69	0.05	+1.96	−1.60
Ni terminal	1.74	0.02	+1.30	−1.93	1.06	0.02	+1.41	−1.58
Mn terminal	3.95	0.01	+1.90	−1.96	3.25	0.01	+2.10	−1.62
	O* state				OOH* state			
	$\|m\|$: M	$\|m\|$: O	q: M	q: O	$\|m\|$: M	$\|m\|$: OOH	q: M	q: OOH
Co terminal	1.39	-0.83	+1.60	−0.63	0.70	0.36	+1.51	−1.36
Fe terminal	3.50	0.26	+1.90	−0.96	3.70	0.02	+1.94	−1.57
Ni terminal	1.07	0.79	+1.43	−0.70	1.16	0.10	+1.39	−1.59
Mn terminal	-	-	-	-	-	-	-	-

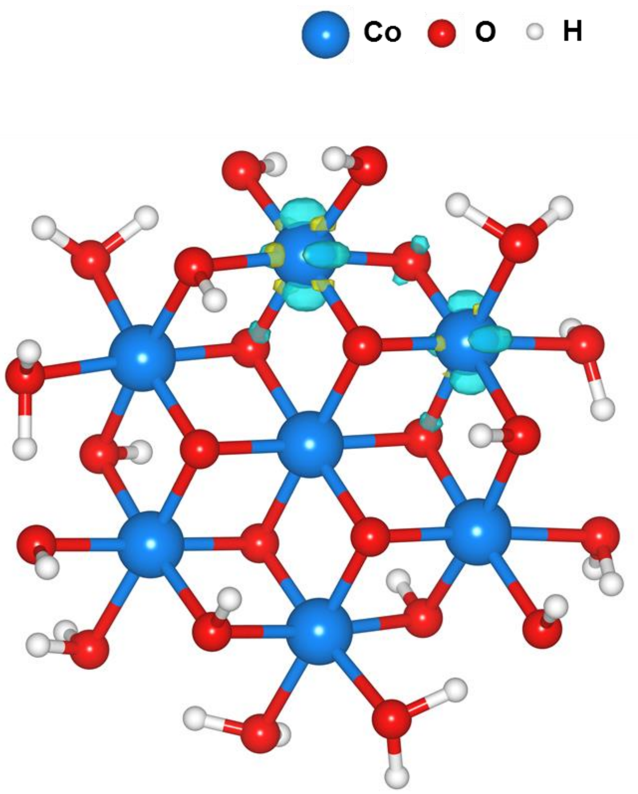

Figure S11. Charge density plot for the bridge site of pristine Co oxide in the O* state, showing the location of holes residing in the two separate Co ions. Blue indicates the charge lost, and yellow indicates the charge gained. The isosurface was set to 0.2 $e^{-}/Å^{3}$.

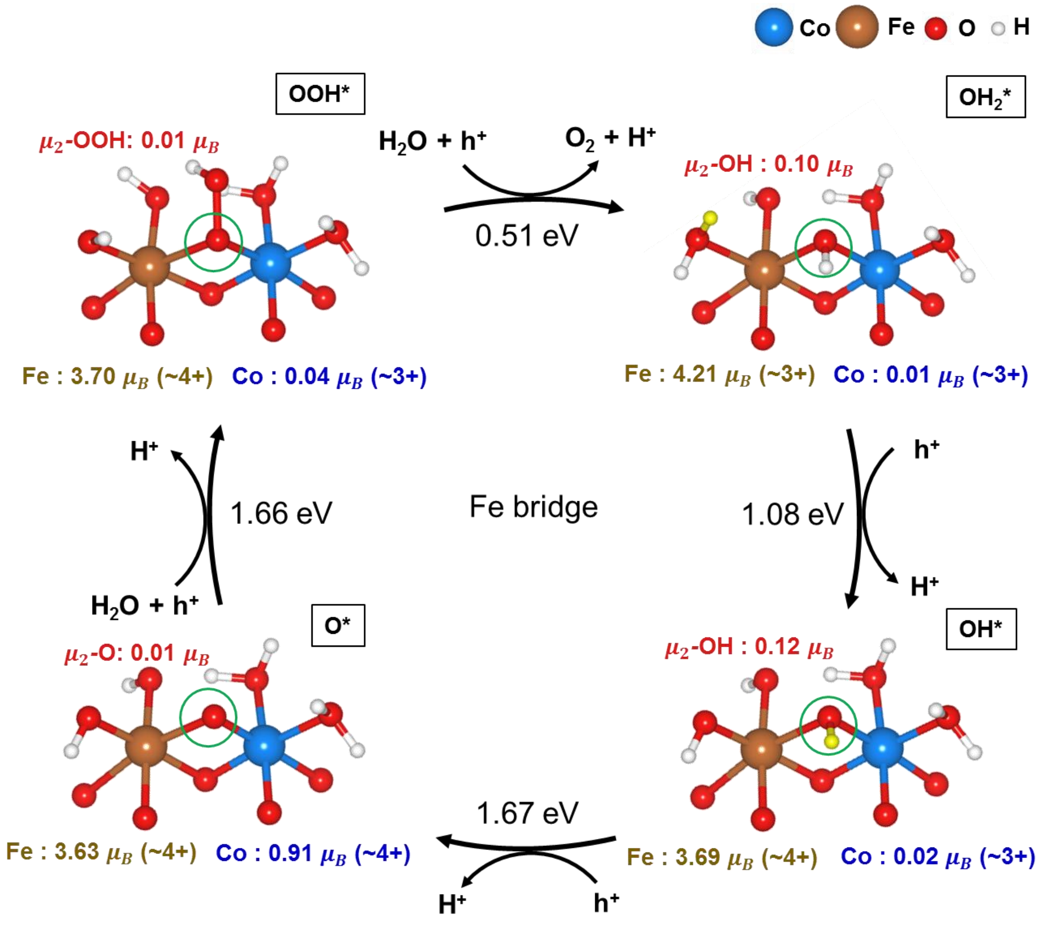

Figure S12. OER cycles for the bridge site of Fe-doped Co oxide. The H atoms to be removed in the next step are colored yellow. The integrated spin moments of the species are also shown in units of electron spin (μ_B).

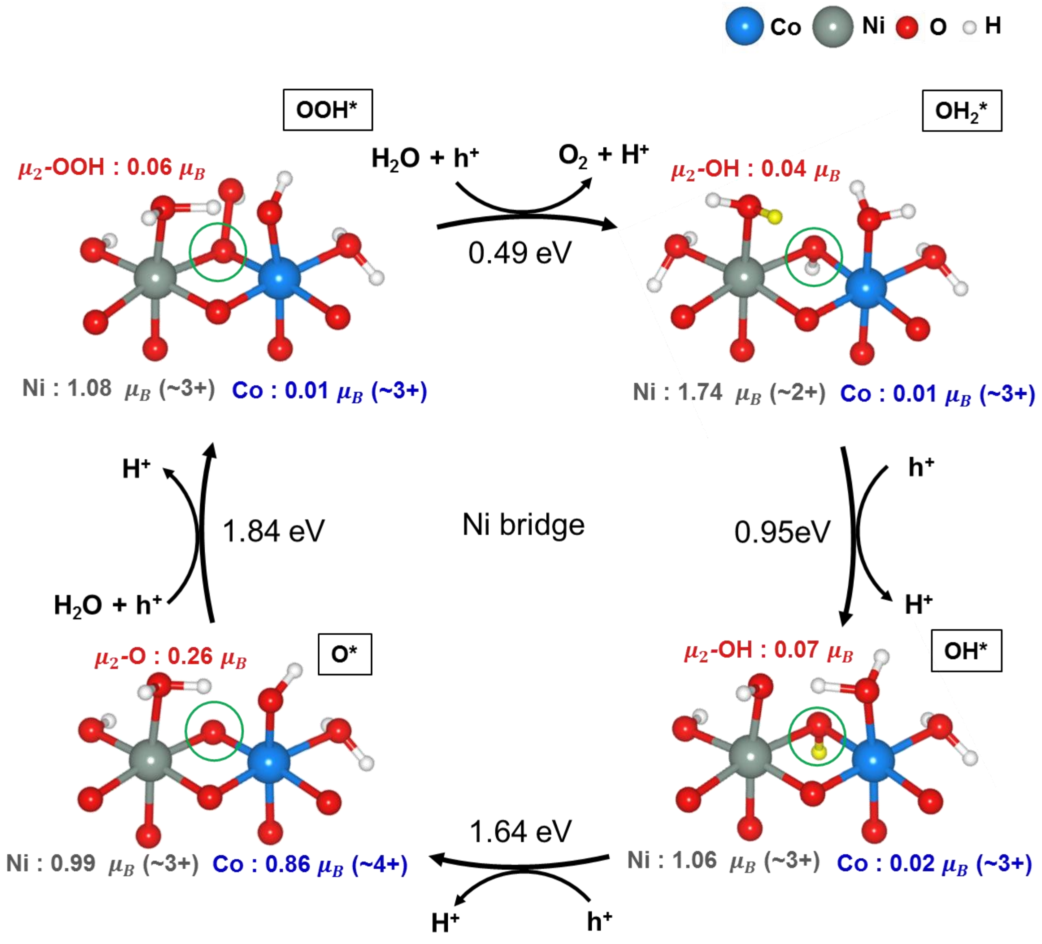

Figure S13. OER cycles for the bridge site of Ni-doped Co oxide. The H atoms to be removed in the next step are colored yellow. The integrated spin moments of the species are also shown in units of electron spin (μ_B).

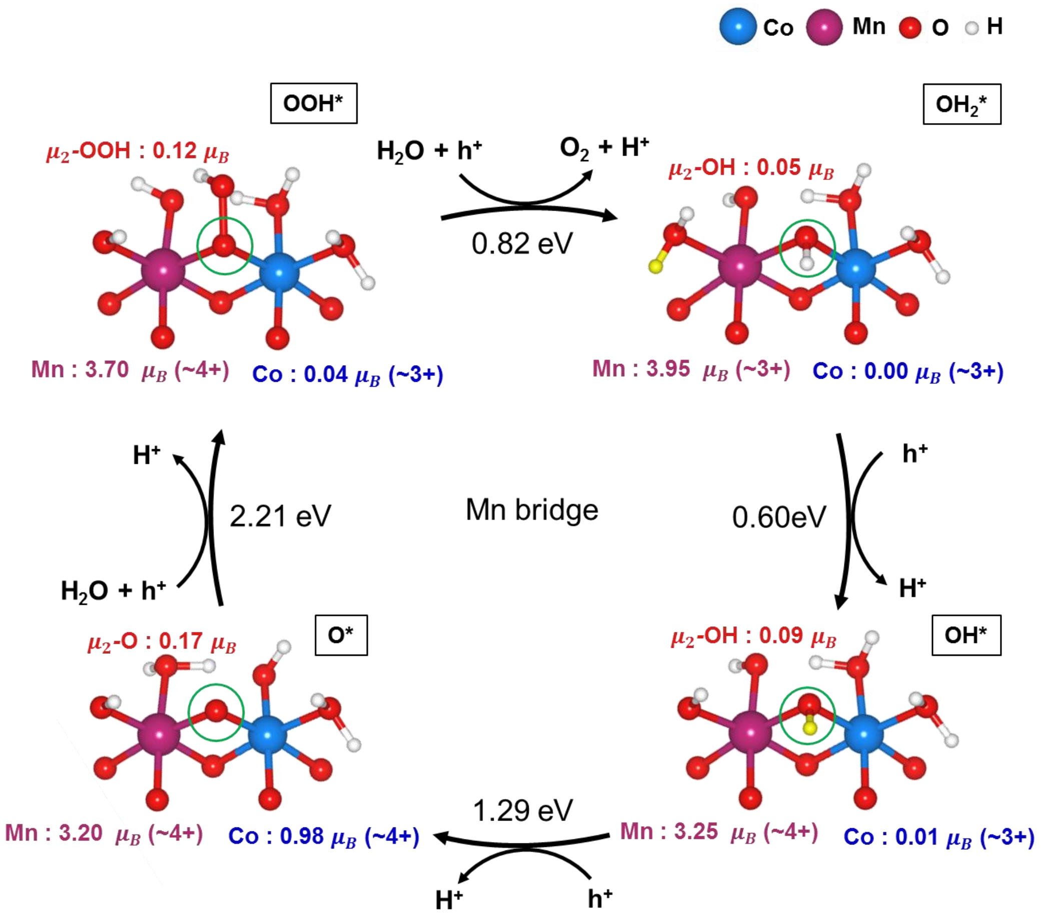

Figure S14. OER cycles for the bridge site of Mn-doped Co oxide. The H atoms to be removed in the next step are colored yellow. The integrated spin moments of the species are also shown in units of electron spin (μ_B).

Table S4. Calculated spin moments m (in units of μ_B) and Bader charges q (in units of Electrons) for two metal ions connected by a bridge oxygen, i.e., M(Co,Fe,Ni,Mn) and Co, and adsorbed species on the bridge sites.

	OH_2* state			OH* state		
	$\|m\|$ (q) : M	$\|m\|$ (q) : Co	$\|m\|$ (q) : μ_2-OH	$\|m\|$ (q) : M	$\|m\|$ (q) : Co	$\|m\|$ (q) : μ_2-OH
Co bridge	0.00(+1.40)	0.00(+1.38)	0.00(−1.51)	1.07(+1.56)	0.00(+1.41)	0.04(−1.46)
Fe bridge	4.21(+1.91)	0.01(+1.40)	0.10(−1.59)	3.69(+1.96)	0.02(+1.42)	0.12(−1.55)
Ni bridge	1.74(+1.30)	0.01(+1.40)	0.04(−1.57)	1.06(+1.41)	0.02(+1.42)	0.07(−1.54)
Mn bridge	3.95(+1.90)	0.00(+1.40)	0.05(−1.61)	3.25(+2.10)	0.01(+1.40)	0.09(−1.52)
	O* state			OOH* state		
	$\|m\|$ (q) : M	$\|m\|$ (q) : Co	$\|m\|$ (q) : μ_2-O	$\|m\|$ (q) : M	$\|m\|$ (q) : Co	$\|m\|$ (q) : μ_2-OOH
Co bridge	1.09(+1.57)	0.84(+1.50)	0.20(−0.77)	1.09(+1.56)	0.00(+1.40)	0.05(−1.51)
Fe bridge	3.63(+1.96)	0.91(+1.53)	0.01(−0.90)	3.70(+1.95)	0..04(+1.41)	0.12(−1.59)
Ni bridge	0.99(+1.41)	0.86(+1.53)	0.26(−0.82)	1.08(+1.40)	0.01(+1.41)	0.06(−1.56)
Mn bridge	3.20(+2.09)	0.98(+1.55)	0.17(−0.91)	3.70(+0.95)	0.04(1.41)	0.12(−1.59)

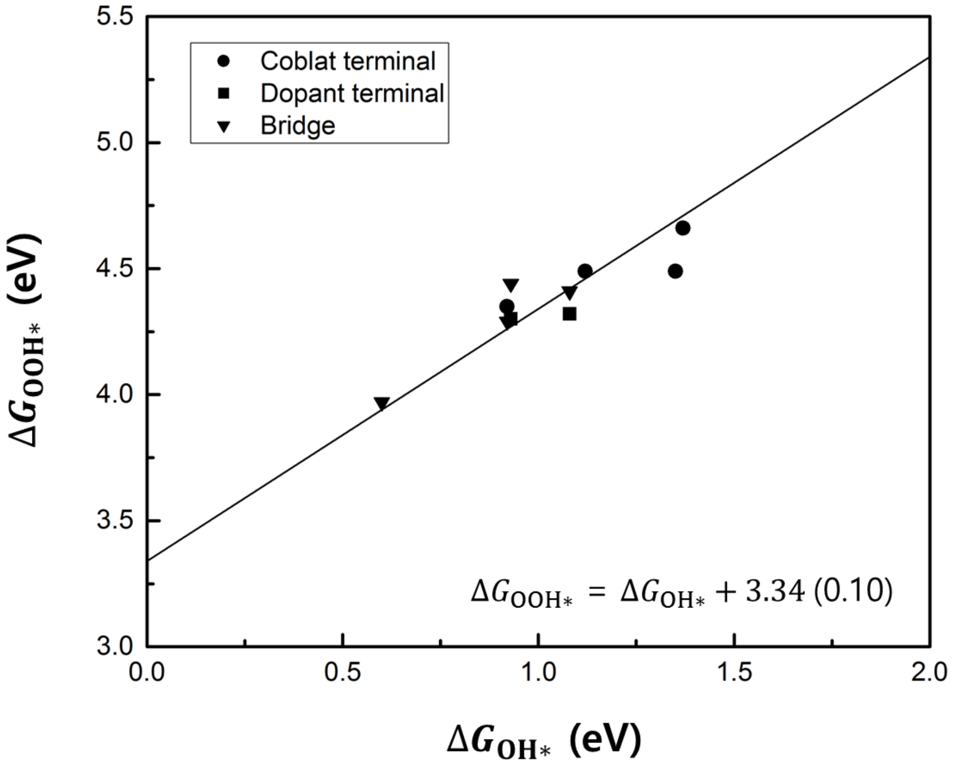

Figure S15. Scaling relations between ΔG_{OOH*} and ΔG_{OH*}. The circle, square, and triangle symbols represent the Gibbs free energy difference calculated for the cobalt terminal, dopant terminal, and bridge sites, respectively. The points are best fit by the linear equation ΔG_{OOH*} = $\Delta G_{OH*} + (3.34 \pm 0.10)$ eV.

The effect of cluster geometry on OER properties

X-ray Absorption Spectroscopy (XAS) analyses revealed that amorphous Co oxides consist of sheets of edge-sharing CoO_6 octahedra.[16] And, it is well-known that Co oxyhydroxides typically crystalize in the plate-like structures in the nature or in the conventional synthetic process.[21-24] However, the precise connectivity between the Co oxide sheets is difficult to be specified due to the amorphous feature of Co oxides. Throughout this work, we applied planar Co_7O_{24} cluster model, which is the most representative model and provides a solid description for the interface between Co oxide sheets and water. If we take the connectivity between Co oxide sheets into consideration, other cluster models having outer-plane motifs in their

strucutres can be possible. On the conditions that models contain the motifs of planar edge-sharing Co oxides, there might be many numbers of cluster models with different connectivitiy between sheets.

For the simplest example, non-flat cluster model can be adopted by considering additional CoO_6 octahedra on the top or at the bottom of the planar cluster to form Co_4O_4 cubane units locally (see Figure S16). For this additional configuration, we calculated OER cycles for the terminal oxygen and bridge oxygen sites of clusters where one CoO_6 octahedron is located above the planar cluster. In good agreements with the results of flat Co_7O_{24} cluster model, the terminal oxygen site in the non-flat model exhibits a low theoretical η of 0.41 V at the same potential-determining (OH* → O*) step. In addition, similar to the bridge oxygen in flat Co_7O_{24} cluster model, the bridge site in non-flat model is predicted to be inactive toward OER because of the large η required for O* → OOH* step. Overall, the presence of the CoO_6 unit on the top of planar cluster is estimated to have insignificant influence on the catalytic activities of nearby reaction sites in the planar motif.

To further understand the effect of cluster geometry in the conditions where dopants are present, we calculated OER pathways for various extended cluster models with dopants. For the planar cluster model in Figure 4, we previously found that the presence of Fe activates the bridge site by reducing the potential of (O* → OOH*) step benefiting from the structural distortions of Jahn–Teller-active Fe(IV) ions. In this respect, we re-investigated whether the beneficial role of Fe ions on the bridge site is valid in the extended cluster models, *i.e.*, clusters with outer-plane CoO_6 octahedra. Figure S17 represents the OER cycles for the cluster where outer-plane CoO_6 octahedra are connected with the planar Co_6FeO_{24} cluster. One CoO_6 unit on one side, or two CoO_6 units on both sides are connected with the planr cluster in Figure S17a and S17b, respectively. For both cases, similar OER pathways and energetics with planar cluster model were obtained. The potential of OOH* formation step reduces to 1.51 eV in both case, resulting in a low η of 0.52 V and 0.58 V in each case. We

further examined the structural properties of these extended clusters during (O* → OOH*) step. In accordance with the observations from the planar cluster, the presence of Fe(IV) ions induces the formation of stronger hydrogen bonding promoting the stabilization of *OOH adsorption (Figure S17d and S17e), in contrast to the case of the pristine Co oxide where the strengthening of hydrogen boding is not expected during the same step (Figure S17c). These findings suggest that the beneficial role of Fe facilitating OER by virtue of its inherent local distortion is also valid for some extended cluster models. Moreover, in Figure S18a and S18b, outer-plane Co ions were placed near the reaction bridge sites, forming Co_3FeO_4 cubane and Co_4O_4 cubane with in-plane metal ions, respectively. Theoretical η was estimated to be 0.56 and 0.53 V for each case, whose much lower η values than that of the bridge site in pristine Co oxides indicates that the existence of Fe dopant in these structural environment also expedites OER process. Interestingly, Fe ions in these clusters have a slightly different local environment than Fe ions in the planar cluster (see Figure S18c). When CoO_6 octahedron is present above the planar motifs, the distortion index of Fe(IV)O_6 is altered due to the elongation of Fe-O1 bond, which results from the formation of the cubane structure. Therefore, in such an environment, there may be other structural or electronic origins by which Fe dopants promote OER, which should be the subject of further work.

Obviously, many other ways of the interconnection among oxide sheets can exist in real materials. Except some conditions that local environment of reaction sites are drastically altred due to the connection with other oxide sheets, the findings of the present work based on planar cluster models are found to be valid for other extended cluster models, as demonstrated above. Future work could explore the effect of outer-plane metal ions and the effect of interactions between the sheets more exhaustively. In particular, the catalytic activities of reaction sites bound to outer-plane metal ions itself can also be affected by the presence of foreign cations, which should be also the avenue of the further study.

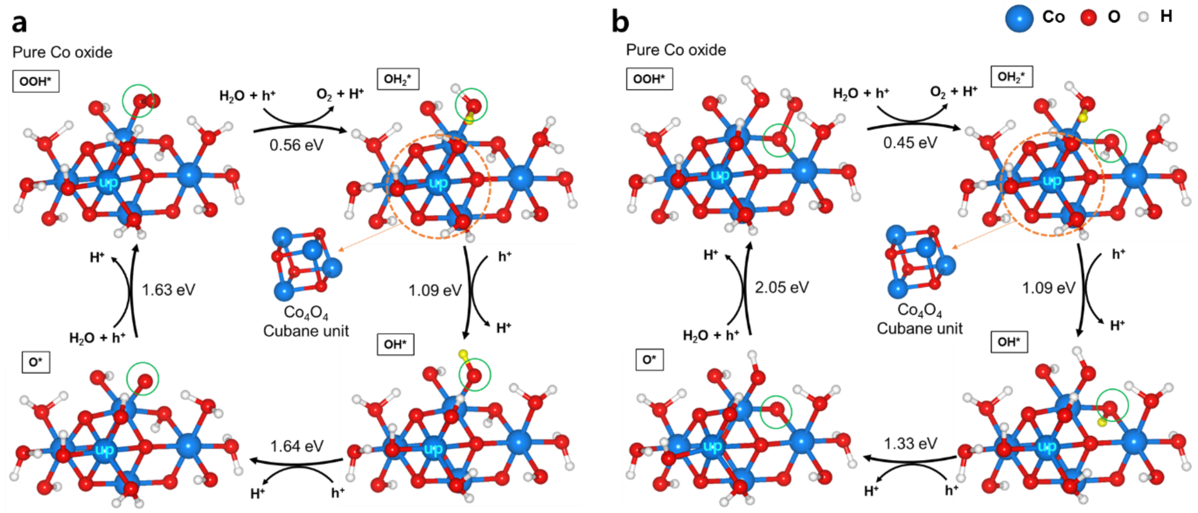

Figure S16. OER cycles predicted for the cluster with the composition $Co_8O_{27}H_{30}$, which consists of planar Co_7O_{24} cluster with CoO_6 octahedron at the top. For the initial state, the cluster is saturated by the appropriate number of H atoms to ensure the valence state of +3 for all Co ions. (a) and (b) indicate OER cycles on the terminal oxygen and bridge oxygen site near the upper Co ion, respectively. The reaction sites are denoted by green circles, and the H atoms to be removed in the next step are colored in yellow.

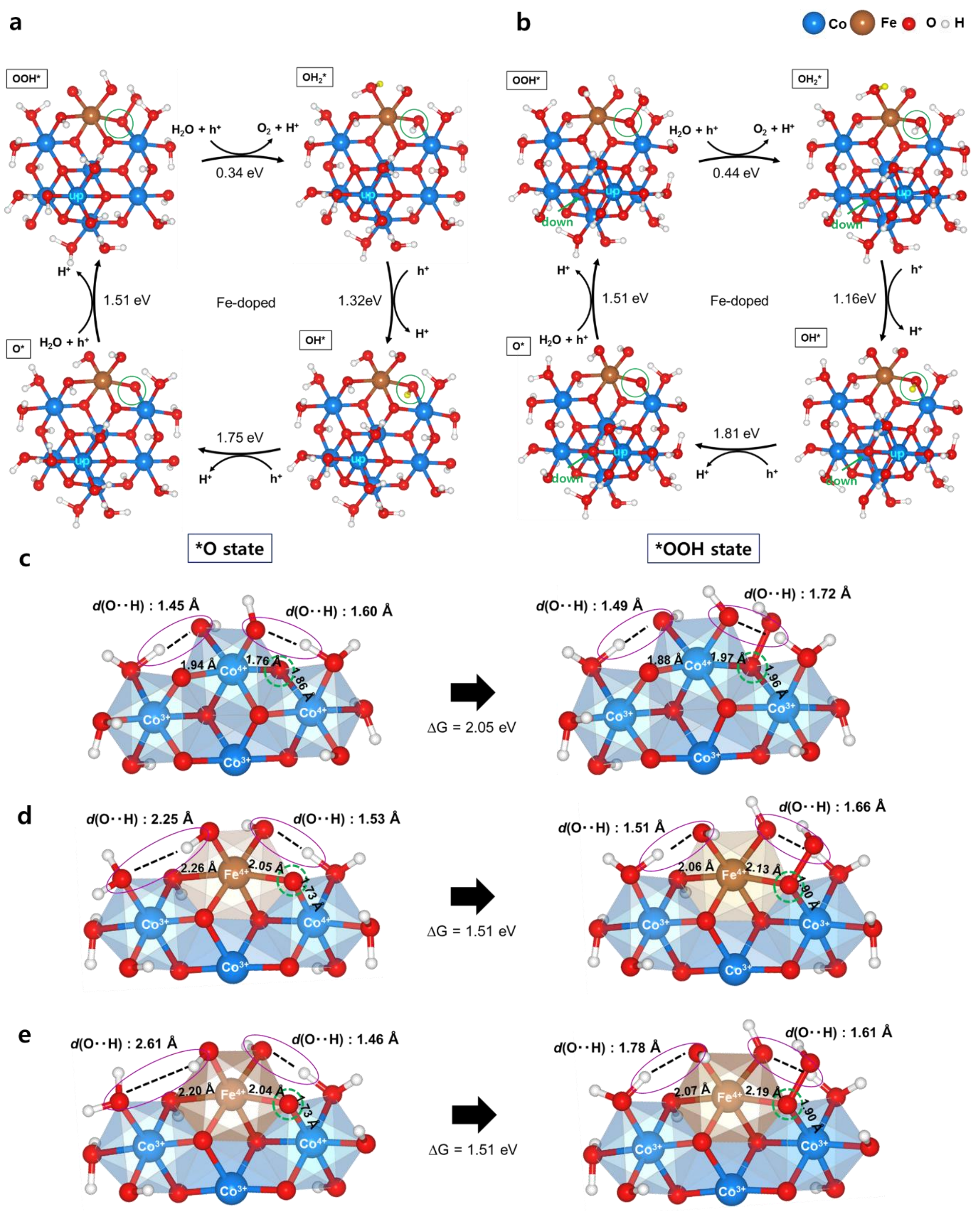

Figure S17. OER cycles for the bridge oxygens sites of models, which consist of planar Co_6FeO_{24} cluster with (a) CoO_6 octahedra on the top ($Co_7FeO_{27}H_{30}$ composition) or (b) CoO_6 octahedra on the top and at the bottom ($Co_8FeO_{30}H_{33}$ composition). For the initial state, the clusters are saturated by the appropriate number of H atoms to ensure the valence state of +3 for all metal ions. The reaction sites are denoted by green circles, and the H atoms to be

removed in the next step are colored in yellow. The comparison of the structures of clusters for the O* → OOH* step is described for the case of (c) the planar Co_7O_{24} cluster with CoO_6 octahedra on the top, (d) the planar Co_6FeO_{24} cluster with CoO_6 octahedra on the top, and (e) the planar Co_6FeO_{24} cluster with CoO_6 octahedra on the top and at the bottom. Only part of the cluster model near the reaction site is described for clarity.

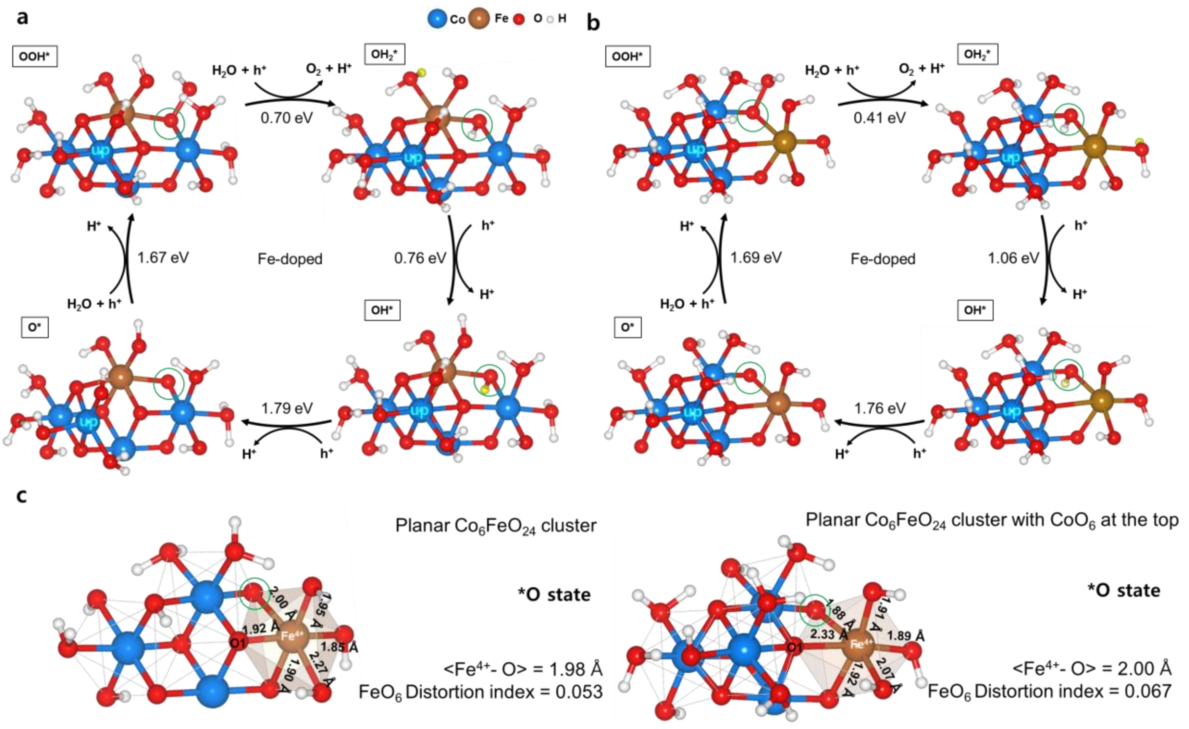

Figure S18. OER cycles at the bridge sites are calculated for $Co_8FeO_{27}H_{30}$ clusters with different configurations of metal ions. (a) Co ion at the top of planar cluster is placed near the reaction site forming Co_3FeO_4 cubane. (b) Co ion at the top of planar cluster is located near the reaction site forming Co_4O_4 cubane. (c) Comparison of structure for the Fe-doped clusters with and without CoO_6 octahedra at the top. Distortion index of FeO_6 octahedra is estimated using Baur's distortion index. [25]

REFERENCES

[1] G. Kresse, J. Furthmüller, *Phys. Rev. B.* **1996**, *54*, 11169.

[2] G. Kresse, D. Joubert, *Phys. Rev. B.* **1999**, *59*, 1758.

[3] S. L. Dudarev, G. A. Botton, S. Y. Savrasov, C. J. Humphreys, A. P. Sutton, *Phys. Rev. B.* **1998**, *57*, 1505.

[4] B. Hammer, L. B. Hansen, J. K. Nørskov, *Phys. Rev. B.* **1999**, *59*, 7413.

[5] Y.-F. Li, A. Selloni, *ACS Catal.* **2014**, *4*, 1148.

[6] F. Costanzo, *Phys. Chem. Chem. Phys.* **2016**, *18*, 7490.

[7] M. Capdevila-Cortada, Z. Łodziana, N. López, *ACS Catal.* **2016**, *6*, 8370.

[8] J. A. Gauthier, C. F. Dickens, L. D. Chen, A. D. Doyle, J. K. Nørskov, *J. Phys. Chem. C.* **2017**, *121*, 11455.

[9] F. Calle-Vallejo, A. Krabbe, J. M. Garcia-Lastra, *Chem. Sci.* **2017**, *8*, 124.

[10] K. Mathew, R. Sundararaman, K. Letchworth-Weaver, T. A. Arias, R. G. Hennig, *J. Chem. Phys.* **2014**, *140*, 084106.

[11] J. Rossmeisl, Z. W. Qu, H. Zhu, G. J. Kroes, J. K. Nørskov, *J. Electroanal. Chem.* **2007**,
607, 83

[12] I. C. Man, H.-Y. Su, F. Calle-Vallejo, H. A. Hansen, J. I. Martínez, N. G. Inoglu, J. Kitchin, T. F. Jaramillo, J. K. Nørskov, J. Rossmeisl, *ChemCatChem* **2011**, *3*, 1159.

[13] J. K. Nørskov, J. Rossmeisl, A. Logadottir, L. Lindqvist, J. R. Kitchin, T. Bligaard, H. Jónsson, *J. Phys. Chem. B.* **2004**, *108*, 17886.

[14] G. Mattioli, M. Risch, A. Amore Bonapasta, H. Dau, L. Guidoni, *Phys. Chem. Chem. Phys.* **2011**, *13*, 15437.

[15] M. W. Kanan, J. Yano, Y. Surendranath, M. Dincă, V. K. Yachandra, D. G. Nocera, *J. Am. Chem. Soc.* **2010**, *132*, 13692

[16] M. Risch, V. Khare, I. Zaharieva, L. Gerencser, P. Chernev, H. Dau, *J. Am. Chem. Soc.* **2009**, *131*, 6936.

[17] J. G. McAlpin, Y. Surendranath, M. Dincă, T. A. Stich, S. A. Stoian, W. H. Casey, D. G. Nocera, R. D. Britt, *J. Am. Chem. Soc.* **2010**, *132*, 6882.

[18] M. S. Burke, M. G. Kast, L. Trotochaud, A. M. Smith, S. W. Boettcher, *J. Am. Chem. Soc.* **2015**, *137*, 3638

[19] F. Song, X. Hu, *J. Am. Chem. Soc.* **2014**, *136*, 16481

[20] L. Trotochaud, J. K. Ranney, K. N. Williams, S. W. Boettcher, *J. Am. Chem. Soc.* **2012**, *134*, 17253.

[21] C. L. Farrow, D. K. Bediako, Y. Surendranath, D.G. Nocera, S. J. L. Billinge, *J. Am. Chem. Soc.* **2013**, *135*, 6403.

[22] Y. Liu, D.G. Nocera, *J. Phys, Chem. C.* **2014**, *118*, 17060.

[23] J. B. Gerken, J. G. McAlpin, J. Y. C. Chen, M. L. Rigsby, W. H. Casey, R. D. Britt, S. S. Stahl, *J. Am. Chem. Soc.* **2011**, *133*, 14431.

[24] Y. M. Chiang, Y. l. Jang, H. Wang, B. Huang, D. R. Sadoway, P. Ye, *J. Electrochem. Soc.* **1998**, *145*, 887.

[25] W. H. Baur, *Acta Crystallogr., Sect. B: Struct. Sci*. **1974**, *30*, 1195.
